# Relationship between root system-soil C:N:P and soil microbial diversity at different evolutionary stages of *Caragana tibetica* scrub in arid desert grassland, Northern China

**DOI:** 10.3389/fpls.2024.1423536

**Published:** 2024-10-16

**Authors:** Min Han, Yumei Liang, Yong Gao, Wenyuan Yang, Yuefeng Guo

**Affiliations:** ^1^ College of Desert Control Science and Engineering, Inner Mongolia Agricultural University, Hohhot, China; ^2^ Key Laboratory of Desert Ecosystem Conservation and Restoration, State Forestry and Grassland Administration of China, Hohhot, China

**Keywords:** *Caragana tibetica* scrub, root structure, root-soil C:N:P, ecochemical measurements, soil microbial diversity

## Abstract

Scrub root systems play a crucial role in preventing soil erosion and nutrient loss. However, the effects of the root system configuration on soil nutrient dynamics and microbial changes at various evolutionary stages remain poorly understood. In this study, we investigated the relationship between soil physical and chemical properties and the diversity of bacteria and fungi throughout the evolutionary stages of the scrub root systems. This was achieved through a combination of whole-root excavation and root tracing techniques. The results indicated that root diameter was the main factor contributing to the continuous increase in carbon (C): phosphorus (P) and nitrogen (N): P ratios as the scrub sand pile developed. The soil organic carbon (SOC), total soil nitrogen (TN), and total soil phosphorus (TP) contents in the soil in the four evolutionary stages were the highest during the developmental stage, but the change in TP content was not statistically significant (*P* > 0.05). Partial least squares path modeling and redundancy analysis (RDA) indicated that root system stoichiometric C and N contents were positively correlated with microbial diversity (R^2^ = 0.85). There was no correlation between the evolutionary stage and soil nutrient TN, whereas soil nutrient TN was negatively correlated with microbial diversity (R^2^ = −0.92). These findings elucidate the relationship between the evolutionary stage of root chemical measurement characteristics, soil elements and microorganisms, and their subsequent effects on root elemental composition and microbial diversity. This study enhances the current understanding of plant–soil interactions in desert steppe ecosystems.

## 1 Introduction

Scrub sand piles form the transition zone of desert grasslands. These unique types of biological landforms, also known as plant dunes, play a crucial role in preventing sand movement and stabilizing the soil, thereby mitigating the large-scale desertification of grasslands and maintaining the ecological balance of the region (Nickling and Wolfe, 1994). The formation of a scrub sand pile occurs when wind-deposited materials accumulate around the scrub, blocking and intercepting the flow of wind and sand (Han et al., 2023). As a drought-tolerant species, *Caragana tibetica* contributes significantly to environmental stability and is used in the management and restoration of desert grasslands. Currently, little is known about the various root adaptations exhibited by scrubs at different evolutionary stages, how the root chemical composition changes, and the impact these changes have on soil nutrients and microbial diversity. Further research is required to provide a theoretical basis for the restoration of desert grassland plants and soils.

The root system is an important organ that connects the soil and plants (Dannowski and Block, 2005), providing essential water and nutrients for plant growth. Its morphological characteristics serve as important indicators of the absorption capacity and growth conditions of plant roots (Liu Y. X. et al., 2023). Studying the morphology of plant root conformation is essential for enhancing the efficiency of soil water and nutrient utilization as well as indirectly reflecting the positive response of the root system to environmental changes (Shan et al., 2014). By measuring the morphological characteristics of scrub sand piles and referencing the various evolutionary stages proposed by Tengberg and Chen (1998), which are based on the morphological characteristics of scrub sand piles and soil and vegetation conditions, there are four main stages of the development of scrub sand piles: fledgling, developmental, stabilization, and recessionary stages (Wei et al., 2021). As these evolutionary stages change, the morphological structure of the plant root system is first affected, mainly through an increase in root diameter and root length. These changes also influence the carbon (C), nitrogen (N), and phosphorus (P) content within the plant root system (Han et al., 2020).

Ecological chemical measurement is a scientific study of the balance between energy and chemical elements in ecological processes. This field offers innovative solutions to the challenges of nutrient supply, demand, and cycling within ecosystems (Jiang et al., 2017). C:N:P ratios in plant roots represent fundamental chemical elements that play a crucial role in plant growth, physiological and metabolic activities, and regulatory mechanisms that respond to environmental changes (Yao et al., 2021). Soil serves as a vital source of nutrients for plant growth and development, and the cycling of C:N:P elements within the soil system has become a focus of attention from researchers, particularly in the context of global climate change and its impact on soil, the environment, and ecological chemical measurements (Wang and Yu, 2008). Investigating the changes and interrelationships between plant root systems and soil C:N:P ratios, as well as their ecological chemical measurement ratios at various evolutionary stages, provides a basis for the study of nutrient cycling in desert grassland ecosystems, as well as the determination of nutrient limitation in vegetated ecosystems.

In this study, we conducted sampling experiments at different evolutionary stages within the sand pile of the Lower *C. tibetica* scrub to investigate the dynamic changes in the chemical measurement and balances of root and soil C:N:P ratios in the arid desert grassland region of northern China. The primary objective of our study was to elucidate the relationships between root system structure, root system, inter-root soil chemical measurements, and inter-root soil microbial diversity. We proposed three hypotheses: (a) root system structure significantly influences the growth and development of scrubs; (b) soil organic carbon (SOC), total soil nitrogen (TN), and total soil phosphorus (TP) content, as well as relative interaction intense (RII) SOC, TN, and TP, reach their maximum values at certain developmental stages; and (c) there is a positive correlation between evolutionary stage and root chemical measurements, and a negative correlation between microbial diversity and soil nutrient levels. To test these hypotheses, we conducted a comprehensive investigation into the effects of root morphology on root chemical measurements, as they vary with the evolutionary stage and are stage-specific for soil nutrients and the presence of bacterial and fungal communities. Based on this, correlations between the root systems of scrub sand piles and soil environmental factors across different evolutionary stages were investigated.

## 2 Materials and methods

### 2.1 Study area

**Figure 1:**
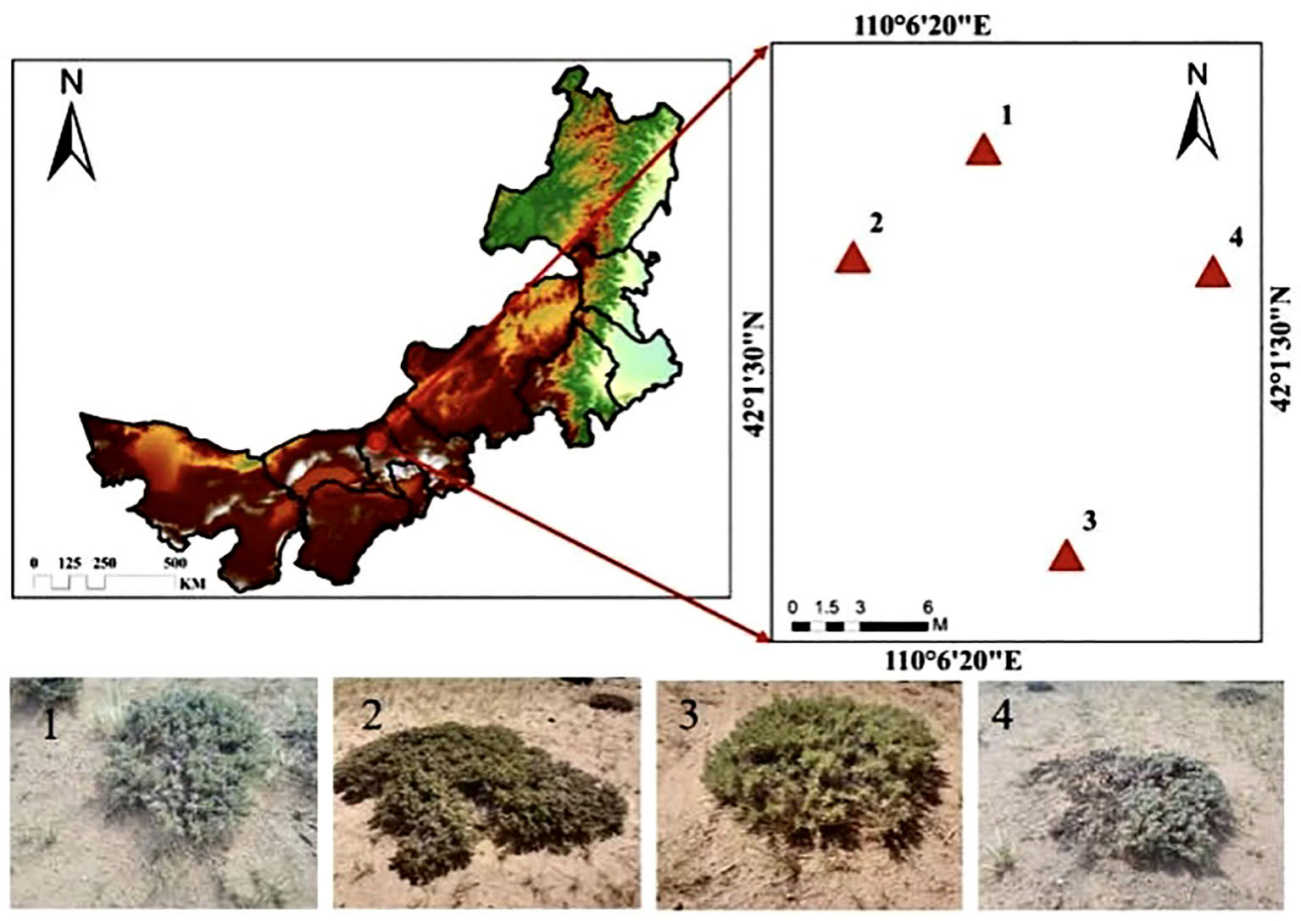
Overview of the study area.

The study area is located near Baiyanhua Gacha, Bailingmiao, Damao Banner, Baotou City. Its geographic coordinates are approximately 110° 06′ 26″–110° 06′ 27″ E, 42° 01′ 49″–42° 01′ 51″ N. The elevation is approximately 1,331 m above sea level, characterized by a topography that is elevated in the southern part of the area and low in the northern of part. The study area has a medium-temperature, semiarid continental climate with a notably uneven distribution of precipitation. This is evidenced by the gradual decrease in annual precipitation from the south to north. The average annual temperature is 5.1°C, with an average annual precipitation of 263.4 mm and an average annual wind speed of 3.0 m·s^−1^ (1991–2020 years). The predominant soil type is typical chestnut-calcium soil, with an effective soil layer thickness of approximately 40 cm, beneath which a calcareous layer is present. The flora in the study area is primarily composed of herbaceous plants, with dominant species including *Leymus chinensis*, *Stipa breviflora*, *Stipa breviflora*, and *Cleistogenes songorica*. The prairie community is simple in structure, with a low grass layer and a sparse structure. The grassland community structure is simple, and the grass layer is low and sparse based on the height of the scrub branches, vegetation cover, and morphological characteristics. The locations of the sample points at different evolutionary stages are shown in **Figure 1**, and the classification criteria are presented in **Table 1**.

**Table 1:** Description of sample plots at different evolutionary stages and morphological characteristics.

Morphological parameters	Longitude and latitude	Altitudes/m	Branch height/cm	Vegetation canopy/cm	Vegetation cover/%	Morphological characteristics
Fledgling stage	110°10′56″E, 42°02′50″N	1333	10.87	28.77	55.92	Monoculture growth, smaller form of scrub sand pile, lower height, and no mortality rate.
Developmental stage	110°10′55″E, 42°02′50″N	1332	13.07	43.62	65.45	Multiple plants growing, densely branched, and in good condition with no mortality and high vegetative cover.
Stabilization stage	110°10′56″E, 42°02′49″N	1331	14.90	106.15	71.22	Multiple growth, dense lateral branching, oval morphology of the scrub sand pile, good plant growth, and relatively high vegetative cover.
Recessionary stage	110°10′57″E, 42°02′50″N	1332	17.73	105.25	58.87	Multiple growth, irregular external morphology of scrub sand piles, large mortality rates in windward slope locations, and low vegetation cover.

### 2.2 Experimental design and methodology

In mid-October 2023, an investigation was conducted on sand piles associated with *C. tibetica* scrubs at different evolutionary stages within the designated study area. A sample plot measuring 100 m × 100 m was established, characterized by the predominance of *C. tibetica* scrub, with no other small plants growing around it, which could potentially influence root growth within the sand pile. Using the method of “space instead of time” (Wei et al., 2021), the representative *C. tibetica* scrub in the sample plot was selected as the research object by combining the relevant literature and the actual situation of the sample plot. The *C. tibetica* scrub was divided into four growth stages: fledgling, developing, stabilizing, and recessionary. Three clumps were selected at each stage for a total of 12 clumps. Measurements were taken and recorded for the long axis, short axis, scrub height, sand pile long axis, short axis, and scrub height. The selected sample plants were excavated using a combination of the full excavation and root tracking methods to investigate the root structure of the entire scrub root system (Yu et al., 2022). The process involved digging at a distance of 1 m from the plant and then carefully removing the soil around the plant using a small shovel. The sandy soil near the root system was continuously cleared until the entire root system >2 mm was completely exposed, and at that point digging was stopped (Zhang et al., 2021). Once the root system was fully exposed, the length, diameter, and number of root connections were measured before and after branching at all levels of the root system using Vernier calipers and tape measures. After recording the data, the collected root systems were transported to the laboratory, and the soil attached to the roots was washed with distilled water. Based on the diameter (d) of the root system, the root system of the thicket was classified into three grades: principal root (d ≥ 10 mm), first-class root (3 mm ≤ d < 10 mm), and secondary root (d < 3 mm) (San et al., 2013) and mounted in different envelopes for labeling.

### 2.3 Sample collection and measurement

#### 2.3.1 Soil sample collection

In the selected study area, a multipoint mixed sampling method was used to collect root soil samples 30 cm–40 cm deep from each evolutionary stage of the thicket. After passing the samples through a 2-mm sieve to remove plant debris and small stones, the soil samples were divided into two parts: one portion was stored in a sterile bag in a refrigerator for bacterial and fungal DNA extraction (Chen et al., 2015), whereas the other portion was placed in a plastic bag for the determination of soil nutrients, such as SOC, TN, and TP.

#### 2.3.2 Root and soil nutrient measurements

The collected root samples were rinsed in the laboratory with tap water one to two times, followed by quick rinsing with distilled water one to two times. The samples were dried in an oven at 105°C for 2 h, followed by drying at 80°C until a constant weight was achieved. After drying, the samples were crushed in a milling machine to pass through a 0.15-mm sieve and subsequently packed in sealed bags for the determination of root C, N, and P contents. Root organic carbon content was determined using the volumetric method with external heating, total nitrogen content was measured using the Kjeldahl nitrogen determination (KND) method, and total phosphorus content was measured using the molybdenum-antimony cliometric method (Ma et al., 2022). SOC content was determined using the potassium dichromate volumetric method, whereas total phosphorus and total nitrogen were detected using the NaOH molybdenum-antimony colorimetric method and the semi-micro KND method, respectively (She et al., 2023).

#### 2.3.3 Microbial high-throughput sequencing

DNA was extracted from soil samples that had been preserved under low-temperature conditions. Total DNA from the extracted soil microorganisms was amplified by polymerase chain reaction (PCR). After amplification, the samples were placed in a foam box filled with dry ice and sent to Shenzhen Microman Technology Corporation for further analysis. The sequencing region of the PCR-amplified standard fungi was the ITS1-5F region, with the upstream primer ITS5-1737F (GGAAGT-AAAGTCGTAACAAGG) and downstream primer ITS2-2043R (GCTGCGTTCTTCATCGATGC) (Yu et al., 2022). The sequencing region of the standard bacterium was the V4–V5 region of the 16S gene, with the upstream primer 515F (GTGCCAGCMGCCGCGGTAA) and downstream primer 907R (CCGTCAATTCCTTTGAGTTT) (Chen et al., 2022). Sequencing was performed using the NovaSeq 6000 PE250 platform (Micromax Technology Ltd.), and data were analyzed using the Micromax Biotech Cloud Platform.

### 2.4 Parameter calculation

#### 2.4.1 Calculation of root diameter ratio of the root system

Root-by-diameter ratio (RBD [i: i + 1]) is defined as the ratio of the roots of class i to the roots of class i + 1 (MaHai et al., 2018), i.e.,


$$RBD\;(i:i+1)=\frac{BDi}{BDi+1}$$

where BD_i_ and BD_i+1_ are the diameters of the branches of levels i and i + 1, respectively.

#### 2.4.2 Topological index

Bouma et al. (2001) and Fitter et al. (1991) proposed two distinct types of root topology: herringbone branching and forked branching patterns. These patterns reflect the branching patterns of scrub root systems at various stages of evolution, as indicated by topological indices. where the topological index expression is:


T1=lgA /lgM

where T1 is the topological index, A is the total number of internal connections within the longest root channel, and M is the total number of external connections associated with the root system. A T1 value closer to 1 indicates that the root system exhibits characteristics similar to herringbone branching and a value of T1 closer to 0.5 represents forked branching. Oppelt et al. (2001) further investigated the topological index configuration on this basis and proposed a new topological parameter. Its expression is given by:


$$q_{a}=\frac{a-1-lbv_{o}}{v_{o}-1-lbv_{o}}$$

$$q_{b}=\frac{b-1-lbv_{o}}{\left(v_{o}+1\right)/2-1/v_{o}-lbv_{o}}$$

where a is the same as A in Fitter’s model, which is the total number of internal connections in the longest root channel, and b is the average topological length, b = Pe/V_0_, where Pe is the total number of all connections in the channel from the base of the root system to the root end. V_0_ is the same as M, the total number of all external connections in the root system, where lbV_0_ = lnV_0_/ln2, where herringbone branching correction q_a_ = q_b_ = 1; forked branching q_a_ = q_b_ = 0.

#### 2.4.3 Root branching rate

The root order was determined according to the methods described by Berntson (1995) and Strahler (1952); that is, the order of roots is determined by the primary root from the inside out. The number of roots (N_i_) at each root level (i) was counted, and the horizontal and vertical coordinates were plotted as grades i and lgN_i_, respectively. The inverse logarithm of the slope of the regression line represents the total root branching rate. The step-by-step branching ratio (SBR) (i:i + 1) is the ratio of the number of branches at level i to the number of branches at the previous level and is calculated using the formula:


SBR (i:i+1)=Ni/(Ni+1)

#### 2.4.4 Calculation of soil nutrient accumulation characteristics

RII was used to express the nutrient enrichment of the sand pile soil (Li et al., 2022b).


$$\text{RII}=\left(\frac{{\text{X}}_{\text{n}}-{\text{X}}_{\text{i}}}{{\text{X}}_{\text{n}}+{\text{X}}_{\text{i}}}\right)$$

where X_n_ and X_i_ represent the soil nutrient content inside and outside the scrub sand pile, respectively.

### 2.5 Statistical analysis

The data collected from the experiment were systematically organized using Excel 2010 software. Statistical analyses were conducted using the SPSS 24 software, and the variability between the different evolutionary stages was assessed using one-way analysis. The results of this analysis were plotted using the Origin software. Additionally, structural variance modeling of root ecological chemical measurements, with soil nutrient and soil microbial diversity indices of *C. tibetica* scrubs at various evolutionary stages, was performed using R language, whereas redundancy analysis (RDA) was performed using Canoco 5 software.

## 3 Results and analysis

### 3.1 Morphological characteristics of scrubs and root systems

The morphological characteristics of the *C. tibetica* scrub and its root system are presented in **Table 2**. The sand pile long axis (Ls), short axis (Ws), height (Hs), horizontal scale (D), and volume (V) of the *C. tibetica* scrub during the decline and stabilization stages were significantly higher than those during the developmental and fledgling stage (*P* < 0.05). However, the ratios of the long axis to the short axis (Ls/Ws) and the sand pile height to the short axis (Hs/Ws) did not show significant differences among the four evolutionary stages (*P* > 0.05). The Ls/Ws values were ranked in descending order as follows: recessionary > developmental > stabilization > fledgling stages. In addition, the values of the sand pile height-to-short axis ratio increased with progression through the evolutionary stages (*P* < 0.05). The sand pile bottom area (A) was significantly higher during the recessionary stage than during the other evolutionary stages, whereas the sand pile height-to-long-axis ratio (Hs/Ls) was significantly higher during the other evolutionary stages than during the fledgling stage (*P* < 0.05). The coefficients of variation (CV) of Hs, Ws, D, A, V, Hs/Ls, and Hs/Ws were greater during the recessionary and stabilization stages than during the fledgling and developmental stages. The coefficients of variation for Ls and Hs/Ls were higher in the fledgling stage than in the other three evolutionary stages, and the CVs for type V were the greatest in the stabilization and decline stages, ranging from 0.46 to 0.67.

**Table 2:** Morphological characteristics of *C. tibetica* scrub at different evolutionary stages.

Morphological parameters	Index	Ls/cm	Ws/cm	Hs/cm	D/cm	A/cm^2^	V/cm^3^	Ls/Ws	Hs/Ls	Hs/Ws
Fledgling stage n=93	Max	28.90	22.30	7.30	25.60	322.24	730.40	1.30	0.41	0.47
	Min	17.60	15.60	6.80	16.60	137.28	331.61	1.10	0.24	0.30
	MS ± SE	21.43 ± 6.48^c^	19.78 ± 3.64^b^	6.86 ± 0.42^b^	20.61 ± 4.58^c^	216.76 ± 95.18^b^	491.89 ± 210.13^b^	1.09 ± 0.24^a^	0.34 ± 0.09^b^	0.36 ± 0.10^a^
	CV	0.30	0.18	0.06	0.22	0.44	0.43	0.22	0.26	0.28
Developmental stage n=148	Max	38.60	35.40	13.40	35.45	628.35	2,806.3	1.40	0.38	0.39
	Min	35.50	27.60	7.60	33.10	532.68	1,397.51	1.27	0.20	0.26
	MS ± SE	37.17 ± 1.56^c^	30.83 ± 4.07^b^	10.60 ± 2.91^b^	34.00 ± 1.27^c^	570.89 ± 50.65^b^	2,040.60 ± 712.56^b^	1.22 ± 0.20^a^	0.29 ± 0.09^ab^	0.34 ± 0.07^a^
	CV	0.04	0.13	0.27	0.04	0.09	0.35	0.17	0.31	0.21
Stabilization stage n=90	Max	116.70	104.50	33.10	110.60	6,097.58	67,276.58	1.21	0.28	0.32
	Min	75.60	62.30	14.30	68.95	2,354.94	11,225.21	1.08	0.19	0.23
	MS ± SE	99.33 ± 21.28^b^	88.37 ± 22.79^a^	25.63 ± 9.98^a^	93.85 ± 21.99^b^	4,549.23 ± 1953.14^b^	43,195.83 ± 28,846.60^ab^	1.14 ± 0.07^a^	0.25 ± 0.05^a^	0.28 ± 0.05^a^
	CV	0.21	0.26	0.39	0.23	0.43	0.67	0.06	0.21	0.17
Recessionary stage n=31	Max	204.50	173.40	26.60	188.95	17,730.15	49,624.52	2.08	0.17	0.36
	Min	147.50	73.40	22.30	112.95	5,596.75	131,794.12	1.00	0.11	0.13
	MS ± SE	168.17 ± 31.56^a^	131.60 ± 51.98^a^	24.13 ± 2.22^a^	149.88 ± 38.04^a^	11,413.97 ± 6,082.07^a^	88,973.16 ± 41,194.70^a^	1.42 ± 0.58^a^	0.15 ± 0.03^a^	0.22 ± 0.13^a^
	CV	0.19	0.39	0.09	0.25	0.53	0.46	0.41	0.22	0.58

Ls, sand pile long axis; Ws, sand pile short axis; Hs, sand pile height; D, sand pile horizontal scale; A, sand pile bottom area; V, sand pile volume; Ls/Ws, ratio of the long axis to the short axis; Hs/Ls, sand pile height to the long axis ratio; Hs/Ws, sand pile height to the short axis ratio; Max, maximum value; Min. minimum value; MS ± SE, mean ± standard error; CV, coefficient of variation. Different lowercase letters indicate differences between different evolutionary stages, as follows.

The frequency distributions of the morphological parameters of *C. tibetica* scrub are presented in **Table 3**. As shown in the Table, the Hs, A, and V, of the *C. tibetica* scrub sand pile were primarily concentrated in the ranges of 0 cm–20 cm, 0 cm–1,000 cm^2^, and 0 cm–10,000 cm^3^ during the fledgling and developmental stages. These parameters accounted for 97.85% and 95.90% of the total volume of the *C. tibetica* scrub sand pile, with values of 95.7% and 91.22%, and 96.75% and 97.30%, respectively. In contrast, Hs and A exhibited greater dispersion during the stabilization and recessionary stages, whereas V was concentrated at ≥30,000 cm^3^, accounting for 96.77% of the total volume. Regarding the height (Hs) of the scrub piles, the number of scrub piles with Hs < 10 cm followed this pattern from the largest to smallest: fledgling stage > development stage > stabilization stage > recessionary stage. For scrub piles with Hs between 10 cm and 20 cm, the pattern was as follows: development stage > stabilization stage > recessionary stage > fledgling stage. In terms of the bottom area (A) and volume (V) of the scrub pile, the number of scrub piles decreased in the following order: A < 1,000 cm^2^ and V < 10,000 cm^3^. The ranking of the scrub sand piles based on the bottom area (A) and volume (V) from largest to smallest was as follows: fledgling > development > stabilization > recessionary.

**Table 3:** Frequency distribution of morphological parameters of *C. tibetica* scrub at different evolutionary stages.

Morphological parameters	Sand pile height		Sand pile bottom area		Sand pile volume	
	Hs/cm	Frequency/%	A/cm^2^	Frequency/%	V/cm^3^	Frequency/%
Fledgling Stage n=93	<10	64.52	<1,000	87.10	<10,000	96.75
	[10,20)	33.33	[1,000,2,000)	8.60	[10,000,20,000)	0
	[20,30)	2.15	[2,000,3,000)	1.10	[20,000,30,000)	1.10
	[30,40)	0	≥3,000	3.20	≥30,000	2.15
Developmental stage n=148	<10	27.70	<1,000	50.00	<10,000	89.19
	[10,20)	68.20	[1,000,2,000)	41.22	[10,000,20,000)	8.11
	[20,30)	4.10	[2,000,3,000)	5.40	[20,000,30,000)	1.35
	[30,40)	0	≥3,000	3.38	≥30,000	1.35
Stabilization stage n=90	<10	20.00	<1,000	35.56	<10,000	70.00
	[10,20)	65.56	[1,000,2,000)	33.33	[10,000,20,000)	26.67
	[20,30)	10.00	[2,000,3,000)	23.33	[20,000,30,000)	1.11
	[30,40)	4.44	≥3,000	7.78	≥30,000	2.22
Recessionary stage n=31	<10	0	<1,000	9.68	<10000	0
	[10,20)	51.61	[1,000,2,000)	12.90	[10,000,20,000)	0
	[20,30)	35.49	[2,000,3,000)	16.13	[20,000,30,000)	3.23
	[30,40)	12.90	≥3,000	61.29	≥30,000	96.77

**Figure 2:**
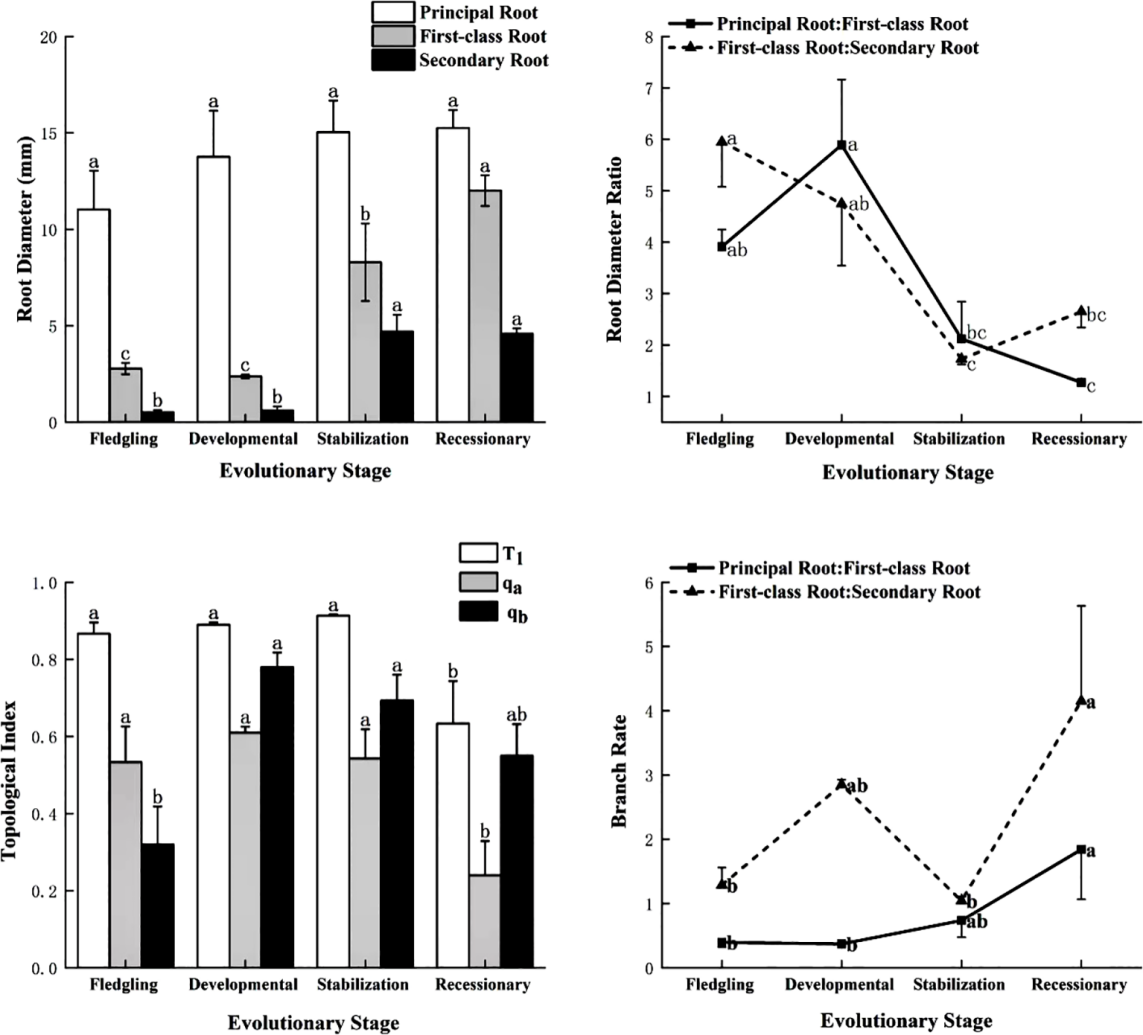
Characteristics of root system configuration of *C. tibetica* scrubs at different evolutionary stages. T1 stands for topological index; qa and qb stand for modified topological parameter calculations.

As shown in **Figure 2**, there was no significant difference (*P* > 0.05) in the diameter of the primary root of the *C. tibetica* scrub at different evolutionary stages, whereas the first-class and secondary roots showed significant differences, with the highest significance (*P* < 0.05) at the recessionary and stabilization stages. The root diameter ratios of the various levels of the root system of the *C. tibetica* scrub at different stages of evolution ranged from 1.27 to 5.95, with the principal and first-class roots showing significant differences in the developmental stage > fledgling stage > stabilization stage > recessionary stage, whereas the root diameter ratios of the first-class and secondary roots were in the following order: fledgling stage > developmental stage > recessionary stage > stabilization stage. The T1 and Q_a_ of the *C. tibetica* scrub root system were significantly similar during the fledgling, developmental, and stabilization stages and less significant in the recessionary stage than in the other three stages. The Q_b_ value was significantly higher during the development and stabilization stages than during the recessionary and fledgling stages. The recessionary stage exhibited forked branches, whereas other evolutionary stages exhibited herringbone branches. The total branching rate of the root systems of *C. tibetica* scrubs was not significantly different (*P* > 0.05), with a branching rate of approximately 2.5. The branching rate of the principal root-first-class root system exhibited significant variability and was significantly higher (*P* < 0.05) during the recessionary stage than during the other evolutionary stages. The branching rates of the first-class and secondary root systems were significant (*P* < 0.05), followed by the recessionary, developmental, stabilization, and fledgling stages.

### 3.2 Characterization of chemical measurements for C:N:P ratios

The C:N:P ratios of the root systems of the *C. tibetica* scrub plants at the four evolutionary stages are presented in **Table 4**. The C and P contents varied within the ranges of 110.85 g/kg to 191.67 g/kg and 0.25 g/kg to 1.07 g/kg, respectively. The levels of C content were observed in the following order: stabilization stage > recessionary stage > development stage > fledgling stage. Conversely, P content exhibited an inverse relationship with C across the various stages in the following order: fledgling, development, stabilization, and recessionary stages. In contrast, the N content varied within the range of 0.77 g/kg to 1.65 g/kg, showing an opposite trend of C, with the order of recessionary stage > development stage > stabilization stage > fledgling stage. The difference between the recessionary and development stages was not significant (*P* > 0.05), but both stages were significantly higher than the stabilization and fledgling stages (*P* < 0.05). The differences among the four evolutionary stages of the C/N ratio were significant (*P* < 0.05), in the order of stabilization stage > fledgling stage > recessionary stage > development stage. In addition, the C/P and N/P ratios increased significantly during the evolutionary stage.

**Table 4:** Descriptive statistics of C:N:P ratio of root systems.

Index	C (g/kg)	N (g/kg)	P (g/kg)	C/N	C/P	N/P
Fledgling stage N = 93	110.85 ± 7.67^b^	0.77 ± 0.07^b^	1.07 ± 0.32^a^	144.16 ± 4.05^b^	108.92 ± 25.17^b^	0.75 ± 0.16^c^
Developmental stage N = 148	112.31 ± 18.91^b^	1.4 ± 0.14^a^	0.8 ± 0.22^a^	81.11 ± 19.02^c^	144.02 ± 26.69^b^	1.86 ± 0.58^c^
Stabilization stage N = 90	191.67 ± 13.72^a^	1.03 ± 0.12^b^	0.29 ± 0.03^b^	188.33 ± 24.01^a^	655.75 ± 52.09^a^	3.58 ± 0.58^b^
Recessionary stage N = 31	169.55 ± 27.50^a^	1.65 ± 0.19^a^	0.25 ± 0.04^b^	104.82 ± 30.2^bc^	681.72 ± 84.81^a^	6.77 ± 1.62^a^

C, root organic carbon; N, root total nitrogen; P, root total phosphorus; C/P, root carbon/phosphorus ratio; C/N, root carbon/nitrogen ratio; N/P, root nitrogen/phosphorus ratio. Changes in C, N and P contents and C/N, C/P and N/P ratios of root systems at different stages of evolution. Different lowercase letters in the graphs indicate significant differences (P< 0.05).

### 3.3 Changes in soil nutrients and microbial diversity in scrub soil

**Figure 3:**
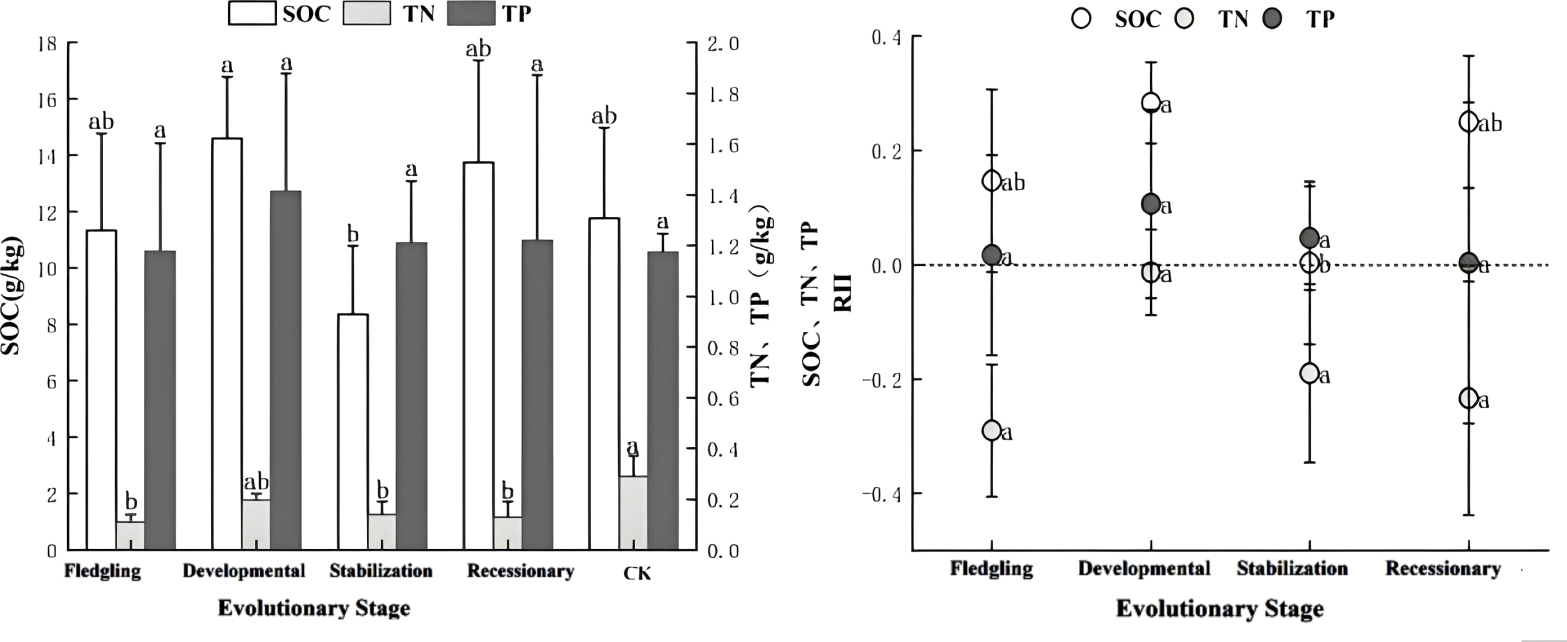
Characteristics of soil nutrient content and accumulation at different evolutionary stages. SOC, soil organic carbon; TN, soil total nitrogen; TP, soil total phosphorus; C:RII, total carbon nutrient accumulation characteristic; N:RII, total nitrogen nutrient accumulation characteristic; P:RII, total phosphorus nutrient accumulation characteristic. Changes in SOC, TN and TP contents and changes in accumulation characteristics at different evolutionary stages. Different lowercase letters indicate significant differences (P< 0.05).

As illustrated in **Figure 3**, the increase in thickness resulted in significant changes in the SOC and TN content (P < 0.05), whereas the TP content was not significant (P > 0.05). The trends for SOC and TP contents were consistent, with the maximum levels observed during the developmental stage. The *C. tibetica* scrub had a significant effect on the soil nutrient accumulation characteristics, as indicated by RII >0 for SOC and TP in the scrub sand pile. This indicated that the nutrient indices exhibited nutrient enrichment at all stages of evolution, with the accumulation effect being more pronounced during the developmental stage. In contrast, the soil nutrient accumulation characteristics for TN across all stages of evolution showed RII <0, indicating a lack of nutrient enrichment, whereas the other indices indicated nutrient enrichment.

**Figure 4:**
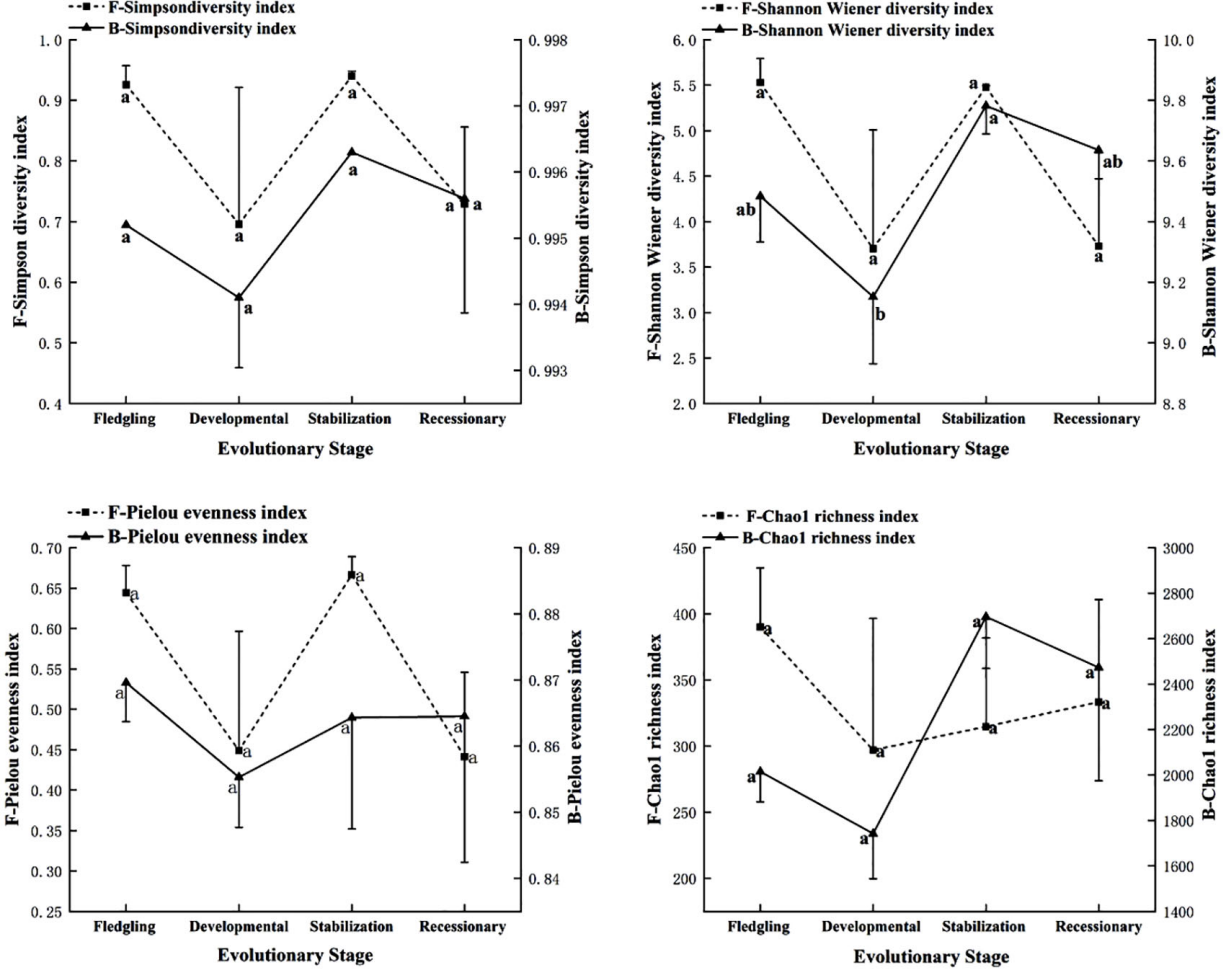
Soil microbial diversity index at different evolutionary stages. F, fungi; B, bacteria. Changes in Simpson diversity index, Shannon-Wiener diversity index, and Pielou evenness index and Chao1 richness index of soil microorganisms, bacteria and fungi at different stages of evolution. Different lowercase letters in the graphs indicate significant differences (P< 0.05).

As shown in **Figure 4**, the Shannon diversity index of soil bacteria within the root system of the *C. tibetica* scrub sand pile was significantly different (*P* < 0.05) compared with that of other evolutionary stages. In contrast, the remaining fungal and bacterial diversity indices were not significantly different (*P* > 0.05). Simpson diversity index, Shannon–Wiener diversity index, and Pielou evenness index of fungi showed that the fledgling and stabilization stages were higher than the developmental and recessionary stages. The Chao1 richness index decreased from the fledgling stage to the developmental stage before increasing again. In contrast to fungal diversity, the Simpson and Shannon–Wiener diversity indices of bacteria showed a similar pattern to that of fungi. However, the Pielou evenness index of the bacteria showed a pattern of decrease, followed by an increase, and then a subsequent decrease across the four evolutionary stages. The Chao1 richness index was the highest during the stabilization stage and the lowest during the developmental stage.

### 3.4 Relationships between root C:N:P ratio, soil nutrients, and microbial diversity

**Figure 5:**
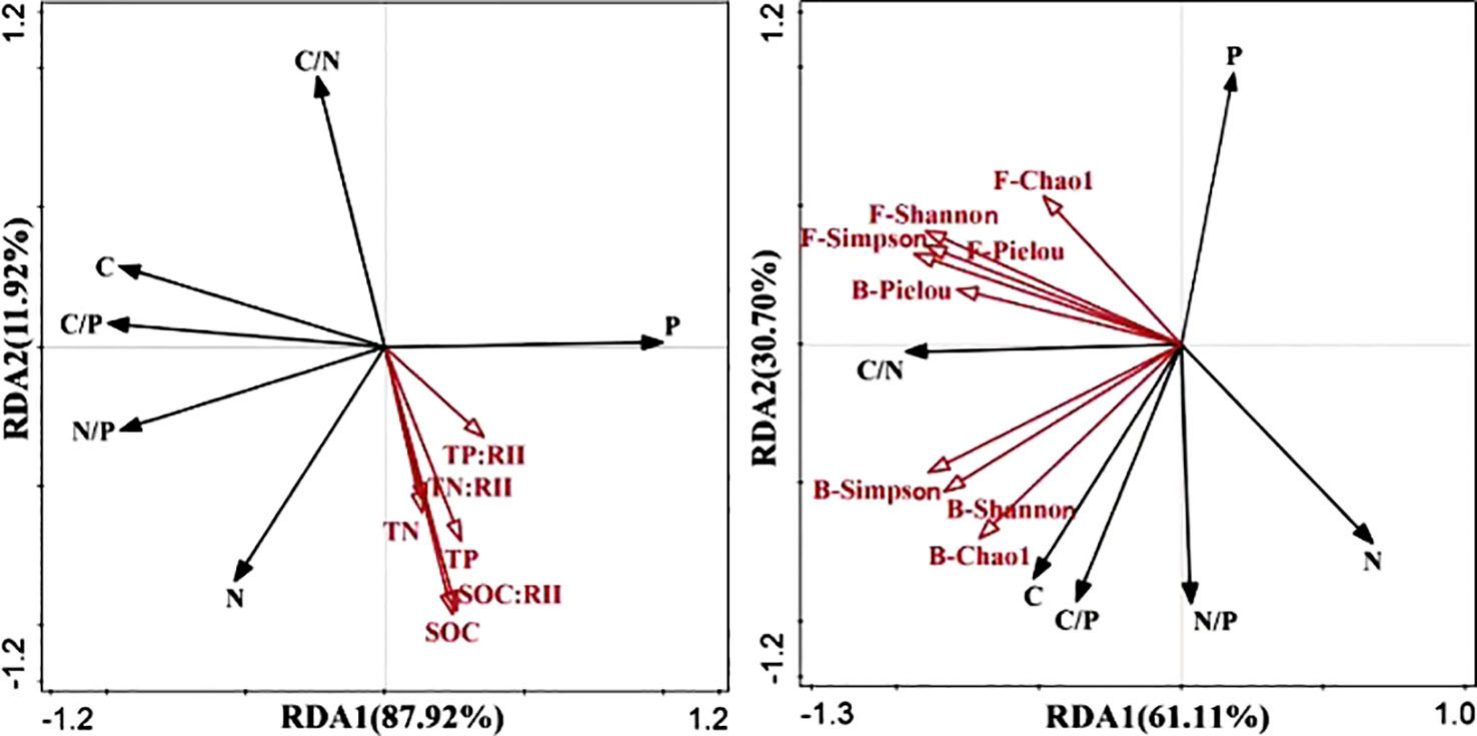
RDA of root chemical measurement traits, soil nutrient levels **(A)**, and soil microbial diversity **(B)** across different evolutionary stages. F, fungi; B, bacteria. C, root organic carbon; N, root total nitrogen; P, root total phosphorus; C/P, root carbon/phosphorus ratio; C/N, root carbon/nitrogen ratio; N/P, root nitrogen/phosphorus ratio; SOC, soil organic carbon; TN, soil total nitrogen; TP, soil total phosphorus; C:RII, total carbon nutrient accumulation characteristic; N:RII, total nitrogen nutrient accumulation characteristic; P:RII, total phosphorus nutrient accumulation characteristic.

RDA revealed that RDA1 and RDA2 explained 87.92% and 11.92% of the total variation in root ecological chemical measurements and soil nutrients, respectively. **Figure 5A** illustrates that the total nitrogen and total phosphorus contents in the roots were positively correlated with soil C, N, and P nutrients, as well as nutrient accumulation characteristics at various developmental stages, with the strongest correlation observed for C. The root N/P ratio was positively correlated with SOC, TN nutrients, and nutrient accumulation characteristics, whereas it was negatively correlated with TP soil nutrients and nutrient accumulation characteristics. Conversely, root C, C/P, and C/N ratios were negatively correlated with soil SOC, TN, and TP, along with nutrient accumulation. Furthermore, RDA was performed to examine the relationship between root chemical measurements and soil microbial diversity and revealed 61.11% and 30.70% of the variation in root chemical characteristics and soil microbial diversity indices, respectively. As shown in **Figure 5B**, the Simpson diversity index, Shannon–Wiener diversity index, Pielou evenness index, and Chao1 were positively correlated with root total phosphorus content and negatively correlated with root total nitrogen content. The root C/N ratio was positively correlated with the four fungal diversity indices and bacterial diversity, indicating that the C/N ratio of the root system was positively correlated with the four fungal diversity indices and the bacterial Pielou evenness index. Notably, the Simpson diversity index of the fungi exhibited the strongest correlation with the C/N ratio of the root system. In addition, the C, C/N, C/P, and N/P ratios of the root system were positively correlated with the other three bacterial diversity indices, whereas they were negatively correlated with the total phosphorus and total nitrogen contents of the root system.

### 3.5 Relative contribution of root C:N:P, soil nutrients, and microbial diversity

**Figure 6:**
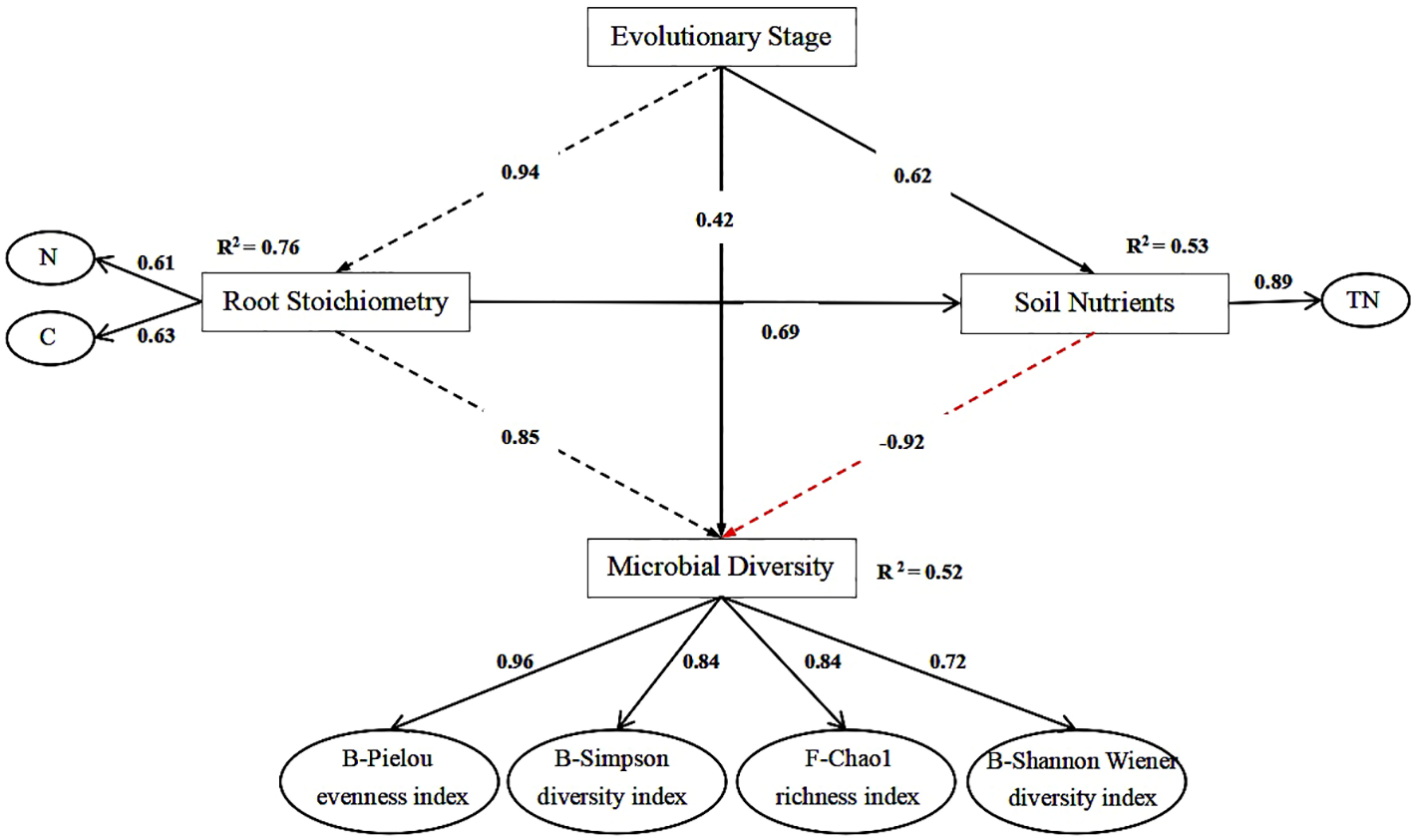
Effect of root chemical measurement on soil nutrient and soil microbial diversity at different evolutionary stages. The black dashed line represents a significant positive correlation, the red dashed line represents a significant negative correlation, the black solid line represents no significant correlation, and the numbers on the arrows represent the path coefficients.

As shown in **Figure 6**, the goodness-of-fit (GoF) value predicted by partial least squares path analysis was 0.63. This value is >0.3 but <0.7, indicating that the model possesses a moderate level of predictive ability. The results indicated that the evolutionary stage was positively correlated with the C and N content of root stoichiometry. Additionally, the C and N contents of root stoichiometry were positively correlated with microbial diversity. The path coefficients between root stoichiometry, soil nutrients, and soil microbial diversity were 0.85 and −0.92, respectively.

## 4 Discussion

### 4.1 Morphological characteristics of scrub root system at different evolutionary stages

The analysis of indicators related to the scrub root system revealed that the morphological characteristics of the root system were influenced by the morphology of the scrub itself, whereas the morphology of the scrub also varied with the root system. It has been reported that scrubs become more developed, and the roots play an increasingly significant role in their development (Guerrero-Campo et al., 2006). As the primary nutrient-absorbing organ, the root system can maximize the absorption of nutrients from the soil, thereby supplying essential nutrients to the plant. The root system uses most resources to develop a large number of fine roots, which leads to variations in fine root diameter and branching rate (Jackson et al., 1996; Kroon et al., 2003). Root morphology shows distinct morphological characteristics with the development of evolutionary stages (Postma and Black, 2021). Notably, the root diameter of the thicket was significantly larger during the decline stage than during the fledgling, developmental, and stabilization stages. This observation suggests that, as the thicket continues to grow, thicker roots are prudently developed to adapt to arid, recessionary environments (Shan et al., 2012). The root diameter ratios of the root systems of *C. tibetica* scrubs at different evolutionary stages were significantly different, with the principal root-first-class root diameter ratio being the most significant at the recessionary stage, and the first-class root-secondary root diameter ratio being the most significant at the fledgling stage; however, in the developmental and stabilization stages, the principal root-first-class root was significantly higher than that of the first-class root-secondary root. According to Li et al. (2022a), the geometric topology of the root system is closely related to the nutrient uptake capacity of soil. They investigated the characteristics of the adventitious root configuration of white *Nitraria tangutorum* scrubs and found that the distribution range of the root system of the forked branch is usually smaller than that of the fish-tail branch, which is unfavorable for nutrient uptake; therefore, the fishtail branch is more adapted to arid and infertile habitats (Fitter, 1987; Fitter and Stickland, 1992; Bouma et al., 2001). In this article, the morphology of the root system during the fledgling, developmental, and stabilization stages exhibited fishtail-shaped branches, and the root system had a larger distribution area, which favored the survival of the thicket. In contrast, during the recessionary stage, the scrubs became more stable and the root system showed forked branches and a reduced root distribution area, leading to a decline in the scrubs. The branching rates of the principal root-first-class and first-class root-secondary roots were the most significant during the recessionary stage, indicating that the root system exhibited a strong expansion capacity at this stage.

### 4.2 C:N:P chemical measurement characteristics of the root system at different stages of evolution

The physiological and ecological functions of scrub root systems are influenced not only by their morphological characteristics but also by their chemical composition (Wu et al., 2023). The C:N:P ratios provide insights into the nutrient demand and utilization efficiency of shrubs, as well as their adaptability to environmental conditions (Liu Y. S. et al., 2023). Through long-term natural selection, plants optimize resource allocation to adapt to the varying environmental conditions, resulting in differences in root chemical composition (Cao et al., 2016). In this study, we found that the C:N:P chemical characteristics of the root system of scrubs were disturbed at different evolutionary stages. The C content of the root system showed the following order: stabilization stage > recessionary stage > development stage > fledgling stage. This was attributed to the high water retention and soil fixation capacity of *C. tibetica* scrubs during the stabilization stage (Jackson et al., 1997), which resulted in a significant increase in the C content of the root system. In the recessionary stage, the root system C content decreased compared with that in the stabilization stage, which was attributed to the death of some scrubs. The root N content is also relatively stable and insensitive to external environmental changes (Yu et al., 2011). The N content of the root system varied significantly among scrubs at different evolutionary stages (*P* < 0.05). The stabilization stage of root P content was low and unresponsive to environmental changes, whereas P content exhibited a decreasing trend across various evolutionary stages. The C/N ratio is indicative of root longevity; a lower C/N ratio in fine roots is associated with a shorter lifespan but increased fine root activity (Chen et al., 2023). C/P and N/P ratios reflect the efficiency of fine root utilization and the rate of plant growth, respectively (Jackson et al., 1997). In this study, the C/N ratio of fine roots was the highest during the stabilization stage. This indicates that fine roots adopt a high-longevity survival strategy at this stage; however, fine root activity decreases, which negatively affects the development of the thicket. The C/P and N/P ratios of fine roots decreased gradually with the growth of the evolutionary stage, indicating that the root system of the scrub rapidly seized spatial resources by accelerating the growth rate during the early stage of growth, whereas the growth activity of fine roots tended to slow or even stagnate when environmental resources became saturated.

### 4.3 Scrub root–soil relationships under different evolutionary stages

Soil is the primary source of nutrients for plant roots, and nearly all the physiological activities of the root system occur in the soil. Soil nutrients and microbial diversity significantly influence the ecological and chemical characteristics of the plant root systems. The results of partial least squares path modeling (PLS-PM) showed positive correlations between evolutionary stages and root chemical measurement, soil nutrients, and soil microbial diversity (R^2^ = 0.76, 0.53, and 0.52), which according to the PLS-PM criteria indicated a high correlation with soil nutrients and a medium correlation with the other two. Scrub is characterized by a well-developed root system. A significant number of compounds from the root layer are deposited in the soil, leading to an increase in the content of SOC, TN, and other components in the root layer soil (Zhang et al., 2023). Analysis of the relationship between the ecological chemical characteristics of the root system in thickets and the soil C:N:P ratio, along with their nutrient accumulation characteristics, revealed that the P content of the root system during the fledgling stage and the N content during the decline stage were positively correlated with the soil nutrient content at the developmental stage. Notably, these correlations were strongest with the soil C content. This finding was consistent with a study by Wang et al. (2023) on the soil carbon content of lemon grassland thicket heaps, which indicated that the C content was higher in the mature stages than in the growth and decline stages.

In arid and semiarid regions of desert grasslands, plant scrub heaps can enhance nutrient requirements by accumulating litter and by other methods. The RDA results for soil microbial diversity indicated that root C content was the primary factor influencing the diversity of the soil bacterial communities. This suggests that most of the bacteria in the study area are microbial taxa associated with carbon sequestration. RDA demonstrated that the stronger correlation between chemical measurements of the scrub root system and bacterial diversity indices resulted from the more direct interaction of soil bacteria with the root-soil (Hannula et al., 2019). Alpha diversity analysis of soil fungal and bacterial communities within the root systems of Tibetan mallard scrubs at various evolutionary stages revealed that the diversity and evenness indices of soil fungi were higher during the fledgling and stabilization stages than during the developmental and recessionary stages. This indicates that the development of Tibetan mallard scrubs created an environment that supported the survival of soil fungi and bacteria in the root system. This aligns with the findings of Wang et al. (2022a, b), who investigated the diversity of soil fungal and bacterial communities in scrub sand mounds at various developmental stages.

## 5 Conclusion

The results of this study indicate that the structural characteristics of the root system influenced its chemical characteristics. Specifically, an increase in root diameter was associated with higher C:P and N:P ratios in the root system. During the developmental stage, the root system exhibited herringbone branching, whereas in the subsequent stages, it exhibited forked branching. This indicates that the developmental stage of the scrub exhibited better growth and increased root branching. The maximum SOC, TN, and TP contents during the developmental stage can be attributed to a greater number of fine roots and elevated inter-root soil nutrient levels. A significant correlation was observed between root carbon content and soil nutrients, with nitrogen content having a significant effect on soil nutrients. The root carbon-to-nitrogen ratio, carbon-to-phosphorus ratio, and organic carbon content had the greatest effects on fungal and bacterial communities. In addition, a negative correlation was found between microbial B-Pielou and soil nutrient TN, whereas a positive correlation existed between microbial B-Pielou and root stoichiometric C and N contents, with correlation coefficients (R^2^) of 0.85 and −0.92, respectively. These findings indicated that the root systems of scrub sand piles have the potential to enhance the soil quality in desert ecosystems and improve the management of desert grasslands. This study contributes to a deeper understanding of soil governance using scrub sand piles in arid desert regions.

## Data Availability

The raw data supporting the conclusions of this article will be made available by the authors, without undue reservation.
